# Antiviral activity of diallyl trisulfide against H9N2 avian influenza virus infection in vitro and in vivo

**DOI:** 10.1186/s12985-021-01641-w

**Published:** 2021-08-19

**Authors:** Le Ming, Zhihui Li, Xiaofang Li, Ling Tang, Guimei He

**Affiliations:** 1 Laboratory of Wildlife Epidemic Diseases, School of Life Sciences, East China Normal University, No. 3663, North Zhongshan Rd, Shanghai, China; 2 Institute of Eco-Chongming (IEC), East China Normal University, Shanghai, China

**Keywords:** Diallyl trisulfide, Antiviral activity, H9N2 AIV, Inflammation

## Abstract

**Background:**

Diallyl trisulfide (DATS) is a garlic-derived organosulfur compound. As it has been shown to have anti-viral activity, we hypothesized that it may alleviate infections caused by H9N2 avian influenza virus (AIV), which is prevalent in poultry with pandemic potential.

**Methods:**

Human lung A549 epithelial cells were treated with three different concentrations of DATS 24 h before (pre-treatment) or one hour after (post-treatment) H9N2 AIV infection. Culture supernatants were collected 24 h and 48 h post-infection and analyzed for viral titers and levels of inflammatory and anti-viral immune responses. For in vivo experiments, BABL/c mice were administered daily by intraperitoneal injection with DATS (30 mg/kg) for 2 weeks starting 1 day after H9N2 AIV infection. Clinical signs, lung pathology, and inflammatory and anti-viral immune responses were assessed 2, 4, and 6 days after infection.

**Results:**

Both pre-treatment and post-treatment of A549 cells with DATS resulted in reduced viral loads, increased expression of anti-viral genes (RIG-I, IRF-3, and interferon-β), and decreased expression of inflammatory cytokines (TNF-α and IL-6). These effects were also observed in H9N2 AIV-infected mice treated with DATS. Such treatment also reduced lung edema and inflammation in mice.

**Conclusions:**

Results suggest that DATS has anti-viral activity against H9N2 AIV and may be used as an alternative treatment for influenza virus infection.

## Background

Influenza is a severe infectious disease of the respiratory tract and contributes to substantial morbidity and mortality. It has been estimated that 290,000–650,000 influenza-associated deaths occur annually [1]. Currently, two classes of anti-influenza virus drugs, M2 channel blockers and neuraminidase inhibitors, are available. Unfortunately, increasing viral resistance, cumulative neurotoxicity, and time-dependent effectiveness of these drugs limit their clinical use [2, 3]. Therefore, novel and more effective anti-influenza drugs are urgently needed.

The anti-viral activity of plant extracts and their derivatives is increasingly being recognized in recent years [4, 5]. More than 40% of modern medicines are derived from plants [6]. Several small molecules extracted from plants have been shown to possess anti-influenza virus activity. For instance, catechins in green tea have significant inhibitory effect on both influenza A and B viruses in vitro [7]. The main active ingredients extracted from dendrobium orchids also have been shown to possess activity against H1N1 and H3N2 influenza viruses in vitro [8].

Garlic has a wide range of biological activities, including anti-fungus, anti-virus, anti-cancer, anti-oxidation, and anti-inflammation [9]. Fresh garlic extracts has been shown to inhibit influenza virus replication [10, 11] and modulate immune and inflammatory responses [12]. In dengue virus infection, an active substance of garlic was shown to reduce inflammatory and oxidative stress responses [13]. Organosulfur compounds are the main effective components of garlic. Among which, diallyl trisulfide (DATS) is much more easily synthesized and stable than others [14]. It has been shown that DATS diminishes NF-κB and TNF-α activity in mice with LPS-induced acute lung injury [15]. However, the role of DATS in immune response against influenza virus infection is not clear.

H9N2 avian influenza virus (AIV) was initially identified in chickens in Guangdong province, China in 1994 [16]. It has widely spread and caused enormous economic loss to poultry industry in China [17, 18]. H9N2 AIV can cross species barriers and have been shown to infect humans [19] and contribute internal genes to H7N9, H5N1, H5N6, and H10N8 influenza viruses that infect humans [19–22]. Therefore, H9N2 AIV has the potential to cause pandemic infections. We hypothesized that DATS can enhance innate immunity against H9N2 AIV infection and tested its anti-viral activity in vitro and in vivo in this study.

## Materials and methods

### Virus

The H9N2 avian influenza virus (A/mallard/Jiangxi/39/2011) stored in our laboratory was isolated from wild duck in Jiangxi Province, China in 2011. The complete sequences of all 8 genomic segments (JQ901621, JQ901632, JQ901643, JQ901654, JQ901665, JQ901676, JQ901687, and JQ901698) of the virus are available from the GenBank. The H9N2 AIV used in this study has been shown to infect and induce acute lung injury in mice [23]. The virus was propagated in 10-day old pathogen free chicken embryos (Ingelheim Vital Biotechnology Co, Ltd, Beijing, China) at 37 °C for 72 h (h). The 50% tissue culture infection dose (TCID50) was determined in human alveolar epithelial cell line A549 cells, and the titer of viral stock was 10^6.9^ TCID50/100 μl.

### Cell culture and reagents

A549 cells were purchased from the Cell Bank Academy of Science (China) and grown in Dulbecco's Minimum Essential Medium (DMEM) (Gibco, USA) supplemented with 10% FBS at 37 °C with 5% CO_2_. MTT (3-[4, 5-dimethylthiazol-2-yl]-2, 5-diphenyltetrazolium bromide) (purity > 98%), amantadine hydrochloride (AMT) (purity > 99%), and DATS (purity > 97%) were purchased from Sigma-Aldrich. DATS was dissolved in 0.1% dimethyl sulfoxide (DMSO; Sigma-Aldrich) at 3 mM as stock solution and stored at − 20 °C before use. AMT was dissolved in 0.1% DMSO at 4 mM and used as positive anti-virus control drug. MTT (5 mg/ml) was dissolved in phosphate buffered saline (PBS) and filtered through a 0.2-µm microporous membrane (Millipore).

### Cytotoxicity assay

The cytotoxicity of DATS and AMT for A549 cells was determined using the MTT assay. A549 cells were seeded at a concentration of 1 × 10^5^ cells/well in a 96-well plate (Corning) and incubated at 37 °C in a 5% CO_2_ incubator for 24 h. When the cells reached 75% confluency, they were replenished with 0.1 ml of maintenance medium (with 2% FBS) containing various concentrations of DATS (75–3000 µM) or AMT (125–4000 µM). Five wells of cell were used for each concentration and incubated at 37 °C in a 5% CO_2_ incubator for 48 h. Cells in wells with 0.1 mL maintenance medium without drugs were used as negative controls. After removal of culture medium in each well, MTT (5 mg/ml, 20 µl) was added and incubated for 4 h at 37 °C. The reaction was stopped by addition of 100 µl of DMSO, and the absorbance (Abs) of the purple formazan formed due to MTT reduction by NAD(P)H-dependent cellular oxidoreductase in live cells was read at 570 nm using a microplate reader (Thermo Fisher Scientific,USA). The percentage of cell viability after drug treatment was calculated as follows: % Cell viability = [Abs of treatment group/Abs of control group] × 100%.

### DATS treatment in A549 cells

The effect of DATS on H9N2 AIV infection was investigated with two different conditions, pre-treatment and post-treatment. A549 cells were seeded in 96-well plates or T-25 culture flasks and grown to 75–90% confluency. For pre-treatment experiments, A549 cells were treated with three different concentrations (375 µM, 187.5 µM and 93.75 µM) of DATS for 24 h, washed twice with PBS, and then infected with H9N2 AIV. For post-treatment experiments, the cells were infected with H9N2 AIV for 1 h, washed twice with PBS, and then incubated in maintenance medium containing three different concentrations (375 µM, 187.5 µM, and 93.75 µM) of DATS. Cells treated with 500 µM AMT were used as positive drug control. To test the effect of DATS on virus adsorption, the cells were inoculated with H9N2 AIV at a multiplicity of infection (MOI) of 0.1 After 1 h of incubation at 37 °C, the cells in wells of a 96-well plate were washed twice with PBS and then incubated in maintenance medium for 48 h. The inhibitory activity of DATS on cytopathic effect (CPE) induced by H9N2 AIV was determined by MTT assay. The cells in T-25 culture flasks were incubated at 37 °C under 5% CO2 with a small amount of maintenance medium, and the supernatants were harvested at 24 h and 48 h after drug treatment and assayed for TCID50 on MDCK cells.

### Quantitative RT-PCR

Quantitative real-time RT-PCR (qRT-PCR) was performed to determine the expression levels of IL-6, TNF-α, RIG-I, IRF-3, IFN-β, and H9N2 AIV M gene. All qRT-PCR primers (Table 1) were designed using the software Primer Premier 5.0 (Premier, Canada). Total RNA was extracted from culture supernatants and cells using the RNeasy Mini Kit (QIAGEN). Synthesis of the first-strand complementary DNA was conducted using the Invitrogen Transcription SuperScript™III RT Kit (Invitrogen, US). Each qRT-PCR reaction contained 10 μL SYBR Premix Ex Taq (2 ×), 0.4 μL forward primer, 0.4 μL reverse primer, 0.4 μL ROX reference dye (50 ×), 2 μL cDNA, 6.8 μL H_2_O (total volume 20 μL), and SYBR Premix Ex Taq RR420A-Tli RNase H Plus (Takara Clontech, Dalian). PCR was performed as follows: 95 °C for 30 s followed by 40 cycles of 95 °C for 5 s and 60 °C for 31 s. Expression levels of various genes were normalized to that of the housekeeping gene GAPDH as its expression level was stable in A549 cells. Four independent PCRs were performed for each sample. All data were analyzed using the Sequence Detector Systems software (Applied Biosystems, USA). Fold change in gene expression was calculated using the 2^−ΔΔCt^ method.

**Table 1:** Primers used in this study

Gene name	Primer sequences for genes in A549 cells (5′–3′)	Primer sequences for genes in mice (5′–3′)
IL-6	F-TCCACAAGCGCCTTCGGTCCAG	F-GAGGATACCACTCCCAACAGACC
	R-CTCAGGGCTGAGATGCCGTCG	R-AAGTGCATCATCGTTGTTCATACA
TNF-α	F-ATGAGCACAGAAAGCATGATC	F-CATCTTCTCAAAATTCGAGTGACAA
	R-TACAGGCTTGTCACTCGAATT	R-TGGGAGTAGACAAGGTACAACCC
RIG-I	F-TCCTTTATGAGTATGTGGGCA	F-CGGTCGCTGATGAAGGCA
	R-TCGGGCACAGAATATCTTTG	R-TACGGACATTTCTGCAGG
IFN-β	F-TGGGACGGGGCTTGAATACTGCCTCCA	F-AGAAAGGACGAACATTCGGAAAT
	R-TCCTTGGCCTTCAGGTAATGCAGA	R-CTTGGATGGCAAAGGCAGTG
GAPDH	F-ATGACCTTGCCCACAGCC	F-TCACCACCATGGAGAAGGC
	R-CCCATCACCATCTTCCAG	R-GCTAAGCAGTTGGTGGTGCA
IRF3	F-TACGTGAGGCATGTGCTGA	/
	R-AGTGGGTGGCTGTTGGAAAT	/
M	F-ATGAGYCTTYTAACCGGGTCGAAACG	/
	R-TGGACAAANCGTCTACGCTGCAG	/

### In vivo experiments

Pathogen free BALB/c female mice (aged 6–8 weeks) were purchased from Shanghai Slake Co, Ltd. (China). To evaluate the effect of DATS on H9N2 AIV-induced lung injury, the mice were randomly divided into infected group, DATS-treated infected group, DATS control group, and uninfected control group (n = 26 mice/group). Mice in infected group and DATS-treated infected group were lightly anaesthetized with diethyl ether and inoculated intranasally with 80 μL of H9N2 AIV in allantois fluid (1 × 10^6^ 50% egg infection dose per 0.1 ml, EID50). Mice in uninfected control group were inoculated with the same dilution and volume of sterile allantois fluid intranasally. One day after virus inoculation, mice were intraperitoneally injected with DATS (30 mg/kg body weight) or 0.9% saline daily for 2 weeks. Five mice from each group were euthanized at days 2, 4, and 6 post-infection to obtain whole lungs, kidneys, spleens, and intestines. The pulmonary index was calculated according to the following formula: Pulmonary index = [Lung weight (g)/Body weight (g)] × 100. A portion of each lung was fixed in formalin and processed for histological examinations as described previously [24]. The other part of each lung was used for viral titration on MDCK cells and expression assessments of inflammatory cytokine genes and H9N2 AIV M gene by qRT-PCR. The viral loads in other organs of infected mice were also determined on MDCK cells at days 2, 4, and 6 post-infection. The remaining mice were monitored daily for clinical signs and body weight for 14 days after infection.

### Statistical analyses

All data are expressed as mean ± standard deviation (SD). Statistical analyses were performed using SPSS for Windows, version 19.0 (SPSS Inc, USA) and GraphPad Prism 8.0 (GraphPad Software, San Diego, CA, USA). The one-way analysis of variance (ANOVA) followed by post-hoc Tukey test or unpaired two-tailed t-test was used to determine the significance of difference between two groups. In all statistical analyses, *p* < 0.05 or *p* < 0.01 was considered significant.

## Results

### Cytotoxicity of DATS

**Fig. 1:**
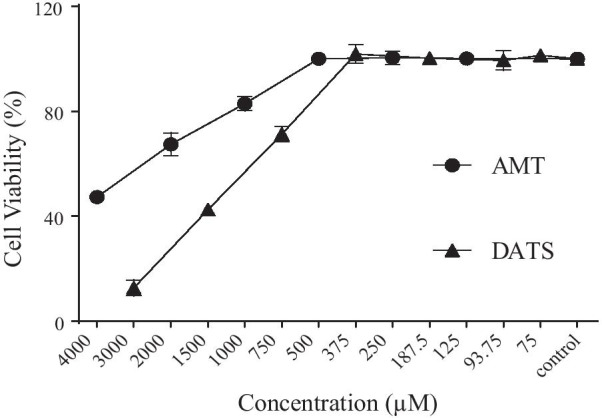
Cytotoxicity of diallyl trisulfide (DATS). A549 cells were incubated with various concentrations of DATS or AMT for 48 h. The percentage of cell viability was calculated. Values are presented as means ± SD of five repeats

The cytotoxicity of DATS and AMT on A549 cells was examined using the MTT-based cell viability assay. Results showed that 375 µM of DATS and 500 µM of AMT had little or no cytotoxicity to A549 cells (Fig. 1). Therefore, 93.75–375 µM of DATS and 500 µM of AMT were used for subsequent experiments.

### Anti-viral activity of DATS

To investigate anti-H9N2 AIV activity of DATS, its effect on different stages of infection was examined. DATS was added to A549 cell cultures to a final concentration of 93.75, 187.5, or 375 µM 24 h before (pre-treatment), or 1 h after (post-treatment) H9N2 AIV infection. H9N2 AIV-infected A549 cells were treated with 500 µM AMT as positive anti-viral control in the same manner. As AMT was dissolved in 0.1% DMSO, cells in untreated control groups were treated with 0.1% DMSO before or after infection to serve as negative controls.

**Fig. 2:**
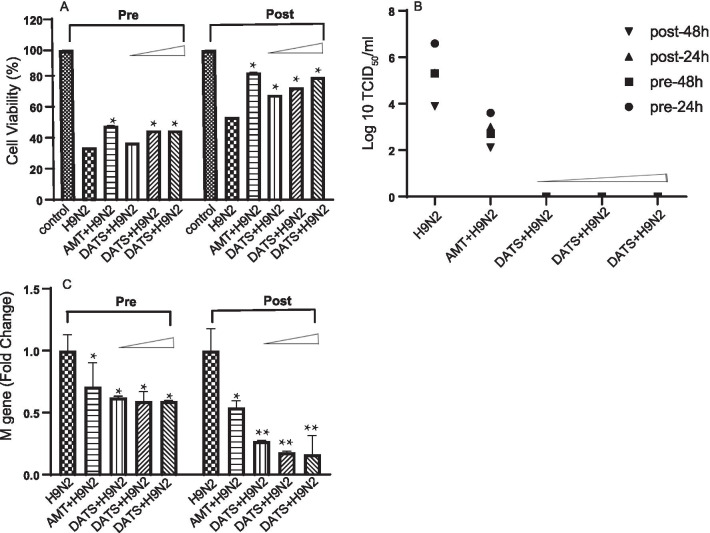
Effect of DATS on H9N2 AIV replication in A549 cells. A549 cells were treated with three different concentrations (375 µM, 187.5 µM, and 93.75 µM) of DATS 24 h before or 1 h after H9N2 AIV infection and then cultured in maintenance medium for 48 h. AMT (500 µM) was used as the positive drug control. **a** Viability of A549 cells was measured by MTT assay. **b** Culture supernatants were collected at 24 h and 48 h after infection and assayed for TCID50 on MDCK cells. **c** RNA was isolated from cell lysates and assayed for expression levels H9N2 AIV M gene by real-time RT-PCR. Abbreviations: control, uninfected cells; H9N2, H9N2 AIV-infected; AMT + H9N2, H9N2 AIV-infected cells treated with 500 μM AMT; DATS + H9N2, H9N2 AIV-infected cells treated with DATS. Values are averages of three independent examinations. Significant difference was determined by comparing the data to those of H9N2 AIV-infected group (*p < 0.05, **p < 0.01)

In the pre-treatment experiment, the viability of untreated H9N2 AIV infected- cells was 33.6%, and that of AMT-treated infected group was 47.8%. The viability of H9N2-infected cells treated with 93.75, 187.5, or 375 µM of DATS was 36.7%, 44.8%, and 44.9%, respectively (Fig. 2a), suggesting that pre-treatment of A549 cells with DATS for 24 h rendered them less susceptible to H9N2 AIV infection. In the post-treatment experiment, the viability of untreated H9N2 AIV infected-cells was 53.5%, and that of AMT-treated infected cells was 82.2%. The viability of infected cells treated with 93.75, 187.5, or 375 µM of DATS 1 h after H9N2 AIV infection was 67.7%, 72.7%, and 79.3%, respectively. The anti-viral activity of DATS was dose dependent and was more effective in the post-treatment experiment.

Culture supernatants of H9N2 AIV-infected cells were assayed at 24 h and 48 h after infection for TCID50 on MDCK cells. Results showed that DATS effectively inhibited virus proliferation in both pre-treatment and post-treatment experiments at 24 h and 48 h (Fig. 2b). To further assess the effect of DATS on H9N2 AIV replication, the expression levels of the M gene of H9N2 AIV in both pre-treatment and post-treatment experiments were examined by qRT-PCR 48 h after infection. Results showed that M gene expression level in AMT pre-treatment experiment was 0.71 fold and that in AMT post-treatment experiment was 0.54 fold of the level of untreated H9N2 AIV-infected cells (Fig. 2c). H9N2 AIV M gene expression in A549 cells in DATS pre-treatment experiment was 0.6 fold (40% decrease) that of untreated H9N2-infected cells for all 3 DATS doses. The inhibitory effect of DATS on H9N2 AIV replication was more profound in the post-treatment experiment as M gene expression in A549 cells treated with 93.75, 187.5, or 375 µM of DATS after infection was 0.27, 0.18, and 0.16 fold that of untreated cells, indicating that DATS at 93.75, 187.5, and 375 µM inhibited H9N2 AIV replication by 73, 82, and 84% when it was added to the cells 1 h after infection. Taken together, these results indicated that DATS was inhibitory to H9N2 AIV replication and was more effective when it was added 1 h after infection. Accordingly, 375 µM of DATS added 1 h post-infection was used for subsequent experiments.

### DATS diminishes H9N2 AIV induced inflammation

**Fig. 3:**
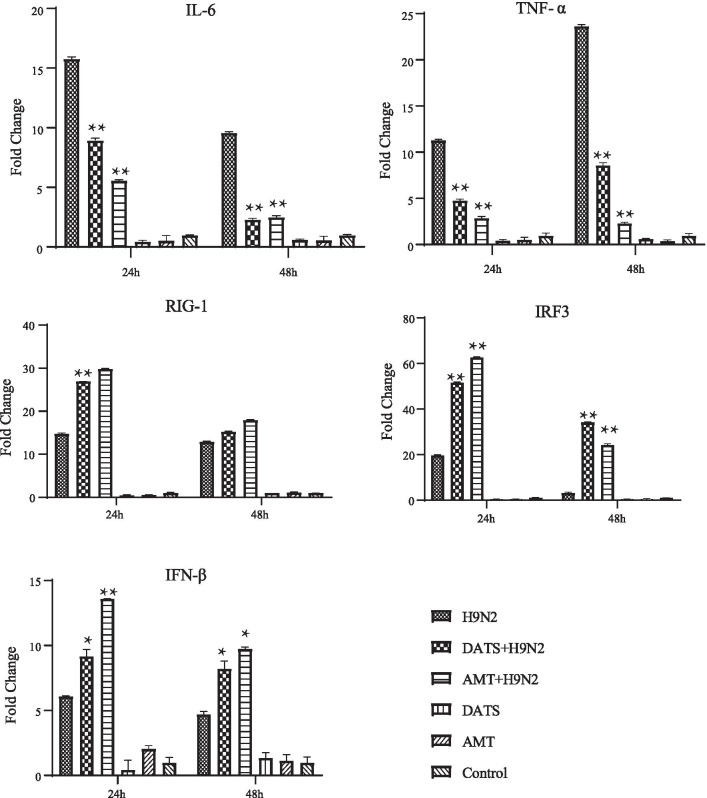
Effect of DATS on the expression of inflammatory and antiviral cytokines. A549 cells were treated with DATS and cultured in maintenance medium. At 24 h and 48 h after DATS treatment, total RNA was extracted and analyzed for expression levels of inflammatory and antiviral cytokines by real-time PCR assay. GAPDH was used as an internal control. Fold change in gene expression was calculated using the 2^−ΔΔCt^ method. AMT (500 µM) was used as the positive drug control. Abbreviations are the same as those in Fig. 2. Values are averages of three independent examinations. Significant difference was determined by comparing the data to those of H9N2 AIV-infected group (*p < 0.05, **p < 0.01)

To investigate the effect of DATS on H9N2 AIV-induced inflammation, the expression levels of inflammatory cytokines TNF-α and IL-6 in cells of post-treatment experiment were measured by qRT-PCR at 24 h and 48 h after infection. The level of TNF-α and IL-6 mRNAs thus determined was then compared to that of uninfected control, which was set as 1.0. For IL-6, H9N2 AIV infection increased its expression by 15.8 fold at 24 h and 9.6 fold at 48 h (Fig. 3a). Treatment of H9N2 infected cells with 500 µM AMT decreased IL-6 expression by 5.6 fold at 24 h and 2.5 fold at 48 h (Fig. 3a). Treatment of H9N2 infected cells with 375 µM of DATS reduced IL-6 expression by 9.0 fold at 24 h and 2.3 fold at 48 h. For TNF-α, H9N2 AIV infection increased its expression by 11.3 fold at 24 h and 23.7 fold at 48 h (Fig. 3b). Treatment of H9N2 infected cells with 500 µM AMT decreased TNF-α expression by 2.9 fold at 24 h and 2.3 fold at 48 h. Treatment of H9N2 infected cells with DATS decreased TNF-α expression by 11.3 fold at 24 h and 4.8 fold at 48 h. These results showed that DATS decreased proinflammatory response induced by H9N2 AIV infection.

### DATS increases anti-viral responses during H9N2 AIV infection

The effect of DATS on the expression of anti-viral factors RIG-I, IRF-3, and IFN-β in A549 cells were also investigated by qRT-PCR. The level of RIG-I, IRF-3, and IFN-β mRNAs thus determined was then compared to that of uninfected control, which was set as 1.0. H9N2 AIV infection was found to increase RIG-1 expression by 14.8 fold at 24 h and 12.9 fold at 48 h post-infection (Fig. 3c). Treatment of A549 cells with 500 µM AMT 1 h after H9N2 AIV infection resulted in a further increase in RIG-1 expression, 29.8 fold at 24 h and 17.9 fold at 48 h. Treatment of cells 1 h after infection with 375 µM of DATS increased RIG-1 expression 26.9 fold at 24 h and 15.3 fold at 48 h (Fig. 3c). For IRF3, H9N2 AIV infection increased its expression by 19.8 fold at 24 h and 3.3 fold at 48 h (Fig. 3d). Treatment of A549 cells 1 h after H9N2 AIV infection with 500 µM AMT increased IRF3 expression 62.8 fold at 24 h and 24.4 fold at 48 h. Treatment of cells with 375 µM of DATS 1 h after infection increased its expression 51.8 fold at 24 h and 34.2 fold at 48 h (Fig. 3d). For IFN-β, H9N2 AIV infection increased its expression by 6.1 fold at 24 h and 4.7 fold at 48 h (Fig. 3e). Treatment of A549 cells 1 h after H9N2 AIV infection with 500 µM AMT increased its expression 13.6 fold at 24 h and 9.7 fold at 48 h. Treatment of cells with DATS 1 h after H9N2 AIV infection increased its expression 9.2 fold at 24 h and 8.2 fold at 48 h (Fig. 3e). These results suggest that DATS exerts its anti-H9N2 AIV by enhancing innate immune responses.

### DATS protects mice from H9N2 AIV infection

**Fig. 4:**
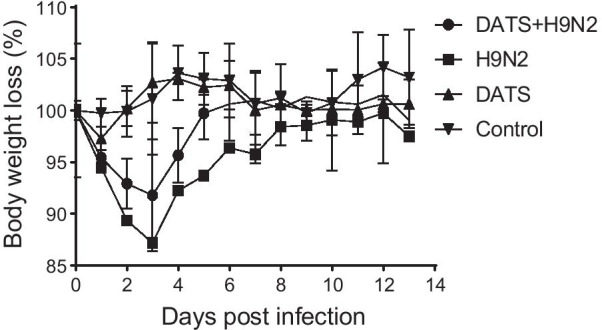
Effect of DATS on body weight of H9N2 AIV-infected mice. BALB/c female mice were inoculated intranasally with 80 μL of allantois fluid containing H9N2 AIV (1 × 10^6^ 50% egg infection dose). One day after virus inoculation, mice were injected with 30 mg/kg of DATS intraperitoneally or 0.9% saline every day and monitored for weight loss daily for 14 days. Abbreviations: Control, uninfected and untreated; DATS, DATS treated uninfected control; H9N2, H9N2 AIV-infected control; DATS + H9N2, mice infected with H9N2 AIV and then treated with DATS. Data are presented as mean ± SD of 6 mice per group

Based on results of in vitro experiments, we hypothesized that DATS can attenuate the symptoms of H9N2 AIV infection in mice. It has been shown that H9N2 AIV-infected mice exhibit marked inactivity, emaciation, ruffled fur, lack of appetite, labored breathing, respiratory distress, and decreased body weight [24]. In this study, the decrease in body weight caused by H9N2 AIV infection was most profound (12.8% loss) 3 days after infection. Treatment of infected mice with DATS (30 mg/kg/day), starting 1 day after infection, diminished such loss (8.2% loss) 3 days after infection. The body weight of mice in DATS-treated group and uninfected control group was not significantly changed (Fig. 4).

**Fig. 5:**
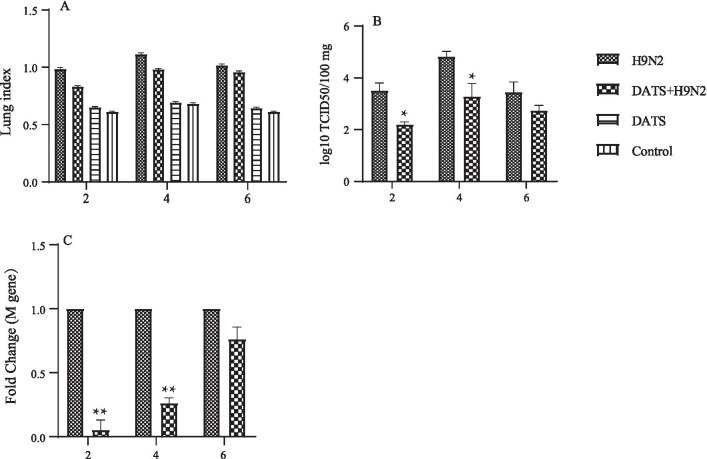
Effect of DATS on lung injury in of mice. Five mice from each group were euthanized at days 2, 4, and 6 post-infection, and their lungs were harvested and weighed. Abbreviations are the same as those in Fig. 4. Significant difference was determined by comparing the data to those of H9N2 AIV-infected group (*p < 0.05, **p < 0.01). **a** Wet lung weight to body weight ratios. **b** Viral loads in the lungs determined by TCID50 assay on MDCK cells. **c** Expression levels of H9N2 AIV M gene determined by real-time PCR. Fold change was calculated using the 2^−ΔΔCt^ method

**Fig. 6:**
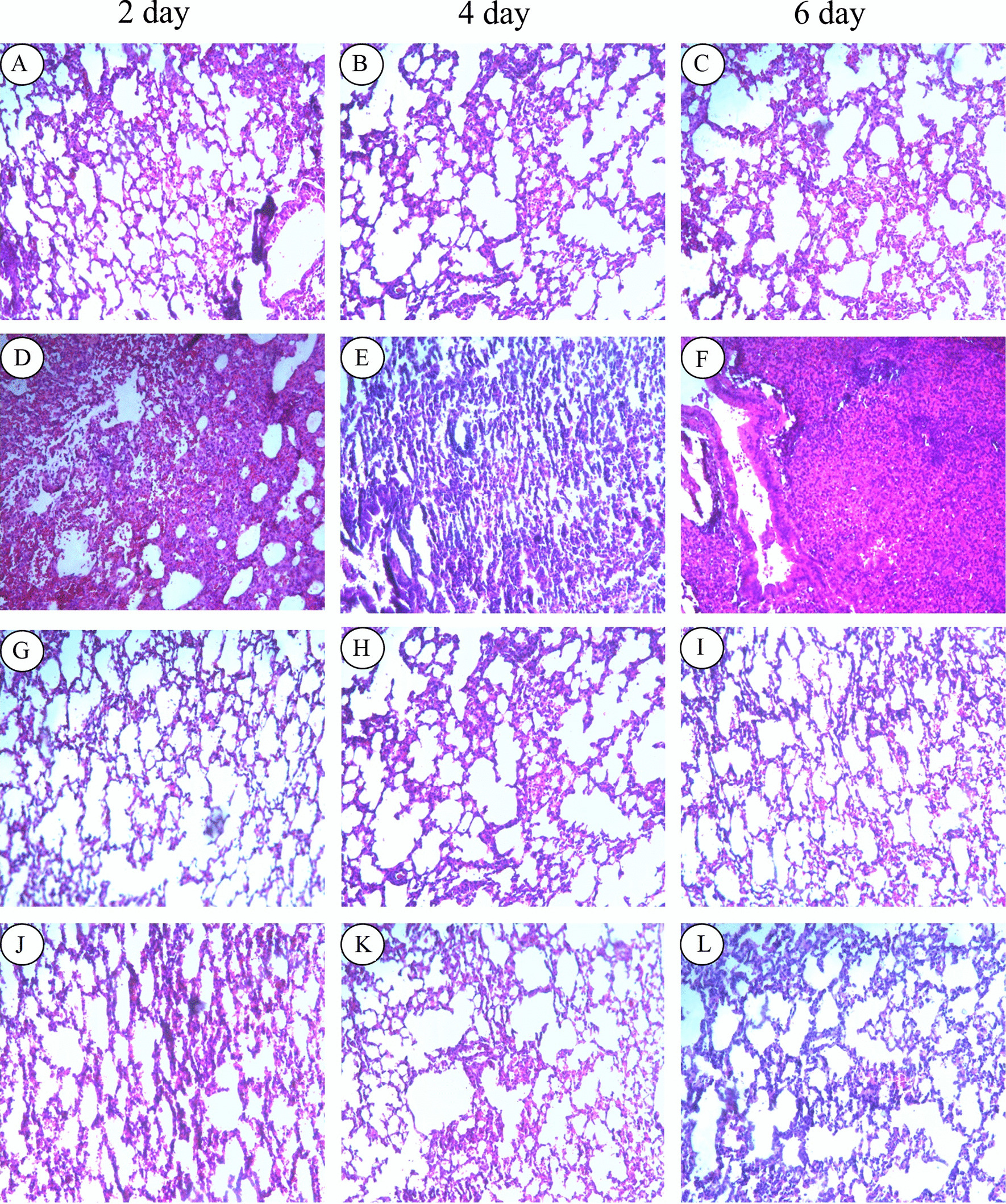
Effect of DATS on lung histology. Five mice from each group were euthanized at days 2, 4, and 6 post-infection, and their lungs were harvested for histological examinations. Abbreviations are the same as those in Fig. 4. DATS + H9N2 (**a**–**c**), H9N2 (**d**–**f**), DATS (**g**–**i**), and Control (**j**–**l**). Images shown are of 100 × magnification (10 × objectives, 10 × eyepiece lens)

To investigate pulmonary edema caused by H9N2 virus infection, lung index (weight lung to body weight ratio) was determined at different time points during infection. Results showed that lung index was increased in mice of infected groups at days 2–6 after H9N2 AIV infection, but it was lower in mice of DATS-treated group (Fig. 5a). At day 6, DATS effect was not significant. These results indicated that H9N2 AIV infection caused edema in the lungs, thus increasing lung index, and DATS treatment reduced the severity of lung edema. Similar patterns of DATS effect were observed in viral loads in the lungs. A significant decrease in viral loads was observed in DATS treatment for 2 and 4 days (Fig. 5b). Since the levels of influenza M protein reflect the levels of virus replication, mRNA levels of the M gene were determined. Results showed significantly reduced levels of M gene mRNA after treatment with DATS for 2 or 4 days (Fig. 5c).To examine lung lesion, HE staining of lung sections was carried out. Results showed that the lungs of H9N2 AIV-infected mice exhibited collapsed alveolar spaces, infiltration of inflammatory cells, interstitial and alveolar edema, and hemorrhage (Fig. 6). These pathological changes were relatively mild in mice with DATS treatment. These results suggest that DATS treatment reduced the severity of edema and viral loads in the lungs.

**Fig. 7:**
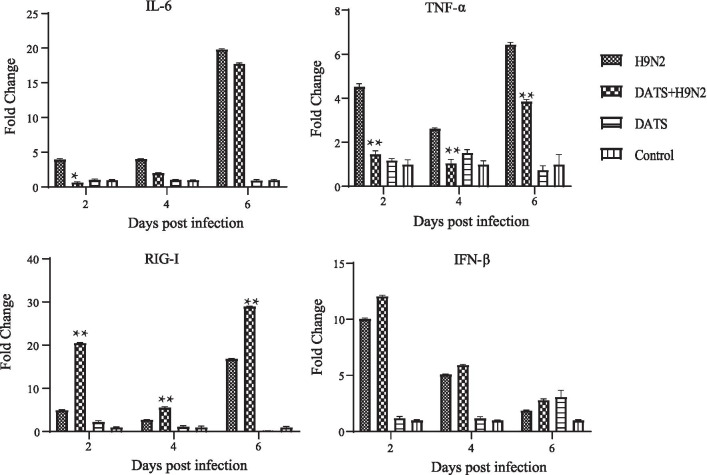
Effect of DATS on the expression of inflammatory and anti-viral cytokines in mice. Five mice from each group were euthanized at days 2, 4, and 6 post-infection, and their lungs were harvested for determination of expression levels of inflammatory and anti-viral cytokines by real-time PCR assay. GAPDH was used as an internal control. Fold change in gene expression was calculated using the 2^−ΔΔCt^ method. Abbreviations are the same as those in Fig. 4. Values are averages of three independent examinations. Significant difference was determined by comparing the data to those of H9N2 AIV-infected group (*p < 0.05, **p < 0.01)

The expression levels of inflammatory cytokines (IL-6 and TNF-α) and anti-viral cytokines (RIG-1 and IFN-β) induce by H9N2 AIV infection in lungs were then determined. The mRNA level of these cytokines was then compared to that of uninfected control, which was set as 1.0. Results showed that the effects of H9N2 AIV infection and DATS treatment on the expression of TNF-α and IL-6 were most profound at 6 days post infection (Fig. 7). At that time, H9N2 AIV infection increased TNF-α expression by 6.5 fold, and DATS treatment reduced it to 3.9 fold. For IL-6, H9N2 AIV infection increased its expression by 20 fold, and DATS treatment reduced it to 18 fold. The effect on RIG-I expression was most profound (16.9 fold increase) at day 6 post-infection. DATS treatment further increased it to 29 fold (Fig. 7). The effect on IFN-β expression was most profound (tenfold increase) at 2 days post-infection. DATS treatment increased IFN-β expression 12 fold. These results showed that H9N2 AIV infection caused a profound increase in the expression of inflammatory and anti-viral cytokines. DATS treatment reduced the expression of TNF-α and IL-6, but increased the expression of RIG-1 and IFN-β.

## Discussion

Influenza viruses infect 3–5 million people every year [1]. Treatment with conventional anti-influenza drugs is usually met with drug resistance. Therefore, alternative anti-viral agents are urgently needed. As garlic and garlic-derived organosulfur compounds have long been used to treat infectious diseases including viral infections [25, 26], we examined the anti-viral effect of DATS on H9N2 AIV infection in vitro and in vivo.

In A549 cells, a strong anti-H9N2 AIV effect was observed when DATS was applied 1 h post-infection (Fig. 2). In the mouse model, DATS was shown to reduce viral loads in the lungs and the severity of lung edema (Figs. 5, 6). This anti-influenza virus activity of DATS is similar to that of fresh garlic extract [10, 11]. It has been shown that over expression of cytokines is a hallmark of severe influenza virus infection [27]. Several reports have demonstrated that the severity and higher mortality of influenza A viral infections were correlated with the excessive inflammation in the lungs attributed to IL-6 and TNF-a [28]. H9N2 viruses are known to elicit a higher expression of inflammatory chemokines and cytokines which might enhance their pathogenicity to the hosts [29]. Garlic has been shown to be effective to several diseases, and which largely due to the reduction of inflammation, and DATS has also been shown to have immunomodulatory and anti-inflammatory effects in several types of cancer [30]. Our data showed that DATS treatment decreased the expression of inflammatory cytokines TNF-α and IL-6 induced by H9N2 AIV infection both in vitro and in vivo.

It has been demonstrated that cytosolic RNAs derived from viral genome are mainly recognized by RNA helicases RIG-I and MDA5 encoded by retinoic acid-inducible gene I (RIG-I) and melanoma differentiation-associated gene 5 (MDA5). RIG-I and MDA5 recruit virus-induced signaling adaptor (VISA) (also known as MAVS, IPS-1, and Cardif) [31]. VISA then forms a large prion-like complex and serves as a platform for the assembly of a signalosome, which contains multiple components including TRAF proteins (TRAF2/3/5/6), TBK1, and IKKs kinases. TBK1 and IKKs phosphorylate IRF3 and NF-B, respectively, leading to induction of type I interferons (IFNs) and pro-inflammatory cytokines [32]. RIG-I is expressed in many types of cells, such as lung epithelial cells, endothelial cells, and fibroblasts and plays a vital role in innate immunity against influenza virus infection. In this study, we showed that DATS up-regulated the expression of RIG-I in H9N2 AIV infected cells, thus promoting the expression of its downstream genes, IRF-3 and IFN-β (Fig. 3).

Results of our in vivo experiments showed that treatment of infected mice with DATS resulted in reduced weight loss, lung damage, and pulmonary inflammation and edema. Pathological examinations revealed that DATS decreased the infiltration of inflammatory cells such as polymorphonuclear neutrophils and macrophages that are important sources of reactive oxygen species (ROS) [33]. Excessive ROS is known to cause oxidative stress, which aggravates the symptoms of viral infections [34]. Whether DATS can diminish H9N2 AIV-induced oxidative stress remains to be investigated. Our results revealed that DATS significantly reduced the expression of IL-6 and TNF-α in H9N2 AIV-infected cells and enhanced the expression of RIG-I and IFN-β in the lungs to defend H9N2 AIV infection in mice. These observations suggest that DATS has the potential to become an alternative therapy for respiratory viral infections.

## Conclusion

We found that DATS inhibited H9N2 AIV infection, increased the expression of anti-viral genes, and decreased the production of inflammation cytokines during H9N2 AIV infection in vitro and in vivo. These results suggest that DATS is a promising antiviral agent against influenza viruses.

## Data Availability

The datasets used in this are available from the corresponding author upon request.

## Abbreviations

DATS: Diallyl trisulfide
AIV: Avian influenza virus
TCID50: 50% Tissue culture infection dose
DMEM: Dulbecco's minimum essential medium
DMSO: Dimethyl sulfoxide
AMT: Amantadine hydrochloride
PBS: Phosphate buffered saline
Abs: Absorbance
SD: Standard deviation
ANOVA: One-way analysis of variance
RIG-I: Retinoic acid-inducible gene I
MDA5: Melanoma differentiation-associated gene 5
VISA: Virus-induced signaling adaptor
